# Medical-Grade Honey Kills Antibiotic-Resistant Bacteria and Prevents Amputation in Diabetics with Infected Ulcers: A Prospective Case Series

**DOI:** 10.3390/antibiotics9090529

**Published:** 2020-08-19

**Authors:** Harikrishna K. R. Nair, Nektarios Tatavilis, Ivana Pospíšilová, Jana Kučerová, Niels A. J. Cremers

**Affiliations:** 1 Wound Care Unit, Department of Internal Medicine, 50586, Kuala Lumpur Hospital, Kuala Lumpur 50586, Malaysia; hulk25@hotmail.com; 2 General Practitioner Xanthi, 67100 Xanthi, Greece; nektart@yahoo.com; 3 Hospital Na Pleši (Nemocnice Na Pleši s. r. o.), Complex Rehabilitation Center, 262 04 Nová Ves pod Pleší, Czech Republic; pospisilova@naplesi.cz; 4 Hospital Na Pleši (Nemocnice Na Pleši s. r. o.), Surgical clinic, 262 04 Nová Ves pod Pleší, Czech Republic; fastkucerka@seznam.cz; 5 Triticum Exploitatie BV, 6222NK Maastricht, The Netherlands

**Keywords:** diabetic ulcers, antibiotic resistance, infections, medical-grade honey, complementary therapies

## Abstract

Diabetic ulcers are at risk of becoming chronic and infected, as diabetics have hampered vascular structures, limiting oxygen and nutrient supply. These wounds can lead to pain, malodor, functional problems, and amputation. The current rise in antibiotic resistance demands for complementary therapies. Medical-grade honey (MGH) forms an attractive option because of its antimicrobial and pro-healing properties. We aim to show the beneficial effects of MGH in infected diabetic ulcers. We present six patients with infected diabetic ulcers, of which some were at risk of (further) amputation. Previous treatments with antibiotics, silver and alginate dressings, surgical closure, and maggot therapy were ineffective; therefore, the treatment was switched to the application of MGH. MGH therapy typically reduced the malodor in a couple of days and controlled infection within 2–3 weeks. MGH also enhanced wound healing by promoting granulation tissue formation, angiogenesis, and re-epithelialization, by decreasing inflammatory and oxidative stress and providing nutrients. Together, wound healing was enhanced, and the patient’s quality of life improved. MGH is safe and cost-effective for treating complicated diabetic wounds with (antibiotic-resistant) infections and at risk of amputation. MGH forms a promising alternative or complementary therapy to replace antibiotics for treating locally infected wounds.

## 1. Introduction

The prevalence of diabetes mellitus (DM) strongly increased over the last few decades [1]. In 2000, the prevalence was estimated to be 171 million, while this number increased to 451 million in 2017 [1,2]. The latter number already exceeds the predictions from 2004, in which 336 million people were predicted to be affected by 2030 [2]. Recent estimations predict a prevalence of 693 million by 2045 [1]. The comorbidities accompanying DM are serious life-threatening health problems that contribute to higher healthcare costs, reduced quality of life for the patient, and higher mortality rates [3]. A common complication is diabetic foot ulcers (DFU), with one-quarter of all diabetics developing one or more DFUs during their life. The definition of a diabetic foot is an “infection, ulceration, or destruction of tissues of the foot associated with neuropathy and/or peripheral artery disease in the lower extremity of a person with (a history of) diabetes mellitus” [4]. DFUs can lead to pain, malodor, functional problems, and eventually amputation when not receiving good care. Especially in diabetics, these ulcers are at risk of becoming chronic and getting infected, as they have hampered vascular structures, limiting oxygen and nutrient supply and transport of leucocytes that are needed to promote wound repair. In addition, DM patients often suffer from neuropathy, which means they cannot feel pain and notice the wounds properly, subsequently resulting in inadequate wound care and exacerbation of the injury. DFUs precede 84% of all diabetes-related lower-leg amputations as they are often contaminated with persistent infections [5]. Forceful drug marketing, competence of medical personnel, and patient compliance are factors contributing to the rise in antimicrobial resistance. The current surge in antibiotic resistance worsens the global outcome of pathogen persistence in infected wounds. Novel therapies working independently of resistance profiles are invaluable. Fortunately, medical-grade honey (MGH) formulations may form a promising complementary therapy that can become more popular in the healthcare sector.

Honey is used for wound healing since ancient times because of its broad-spectrum antimicrobial and wound-healing activities [6]. Antibiotics replaced the use of honey, but the development of antibiotic resistance returned its use [7]. To assure the safety and efficacy of honey for clinical application, strict guidelines are followed to introduce MGH [8]. MGH must be free of any form of contamination, such as herbicides, pesticides, heavy metals, and dormant endospores. MGH must be collected in organic regions and gamma-sterilized, in addition to following strict quality, processing, and storage standards and regulations, in order to ensure the safety of the honey [8,9]. MGH has multiple physicochemical properties that result in antimicrobial and healing activities.

MGH consists of more than 200 different constituents, of which water (17–18%) and carbohydrates (about 80%), such as glucose, fructose, and sucrose, encompass the relative majority. The sugar-rich composition has a hygroscopic activity, attracting fluid from the surrounding environment. This process also leads to dehydration of present microorganisms, making them vulnerable. The acidic pH of MGH makes it even harder for bacteria to persist. The release of the known antimicrobial hydrogen peroxide subsequently kills almost all microorganisms, including those resistant to antibiotics. Hydrogen peroxide is formed by the enzyme glucose oxidase, which the bees add to the honey, and it catalyzes glucose in the presence of water and oxygen into gluconic acid and hydrogen peroxide (C_6_H_12_O_6_ + H_2_O + O_2_ → gluconic oxidase→ C_6_H_12_O_7_ + H_2_O_2_). Moreover, other molecules present in MGH also have a direct antimicrobial effect, such as phenolic compounds, flavonoids, methylglyoxal, and Bee defencin-1 [6]. Since the antimicrobial activity of MGH is based on multiple mechanisms, microorganisms are not capable of developing resistance toward MGH [10]. Interestingly, smart honey formulations have added supplements such as vitamins C and E to further enhance the antimicrobial activity of raw honey [11,12,13]. MGH decreases the bacterial load, while it additionally prevents the invasion of new pathogens by forming a physical barrier when applied to the wound. Together, MGH offers a potent alternative for antibiotics for the treatment of locally infected wounds.

In addition to antimicrobial activity, MGH possesses strong healing activity [7,14]. MGH enhances healing by providing a moist and more regenerative wound environment, being anti-inflammatory and anti-oxidative, and by stimulating autolytic debridement, angiogenesis, and re-epithelialization [7,14,15,16,17]. Moreover, quality of life is improved by reducing pain, minimizing scar formation, and deodorizing wounds [7,18].

Despite all these beneficial effects, the use of MGH is reserved and often limited to later lines of therapy, because clinicians unfortunately tend to stick to conventional treatments such as povidone iodine and antibiotics. Therefore, we aim to raise awareness for MGH as an alternative antimicrobial chemotherapeutic by presenting a case series of infected diabetic ulcers treated with MGH. Most cases were ineffectively treated previously with other therapies, including antibiotics. The use of MGH as an earlier line of treatment can enhance healing and prevent exacerbation of the injuries and potential subsequent amputations. This case series demonstrates the efficacy of MGH against antibiotic-resistant infections and the ease of application that will favor patient and healthcare.

## 2. Results

### 2.1. Case 1

**Figure 1:**
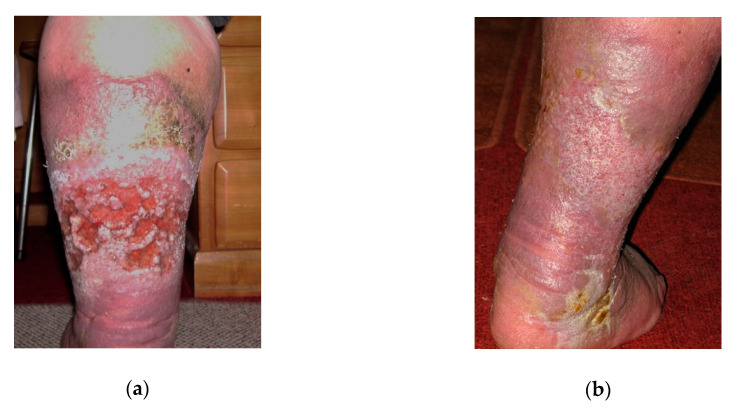
Case 1: (**a**) the wound at the start of medical-grade honey (MGH) therapy (day 0); (**b**) complete healing of the wound after 49 weeks of MGH therapy.

A 78-year-old obese male patient with venous insufficiency and type 2 DM that is non-compliant to his diabetic diet presented to the wound care clinic with an infected leg ulcer. The wound was previously treated with different therapies, including silver sulfadiazine, collagen (Promogran), paraffin gauze (Jelonet), calcium alginate (Algisite), enzyme products (Fibrolan), and silicone sheets (Mepilex), without adequate response. The wound appeared to be infected with ciprofloxacin-resistant *Pseudomonas aeruginosa* and *Streptococci* bacteria (Figure 1a, day 0). At the start of MGH therapy, the legs were edematous, and the wound was painful. A couple of days later, the malodor disappeared, while the infection was resolved within four weeks. Within the same period, pain, edema, and the production of excessive exudate gradually disappeared. Granulation tissue was evident after four weeks, and the wound was completely healed after 52 weeks of MGH therapy (Figure 1b, week 49). The total material costs for L-Mesitran treatment were €159 (4 × 50 g Soft and 2 × 10 pcs Net).

### 2.2. Case 2

**Figure 2:**
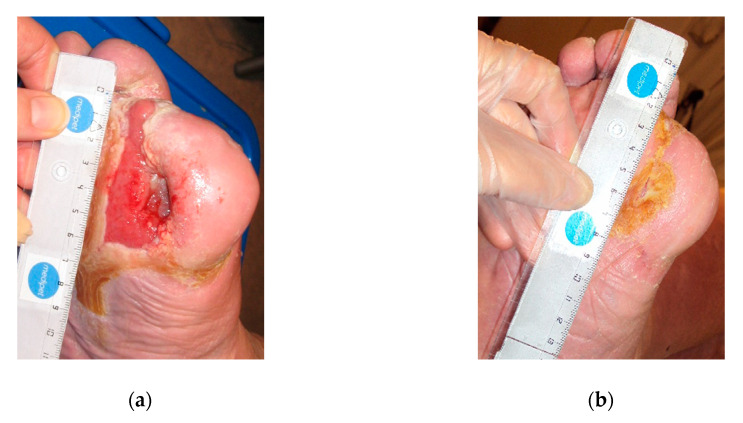
Case 2: (**a**) the wound one week after the start of MGH therapy; (**b**) complete healing of the wound after 34 weeks of MGH therapy.

A 63-year-old obese male patient with type 2 DM presented to the wound clinic with a DFU at risk of amputation for another part of his right foot. Previously, one toe was already amputated because of a non-healing DFU. The current wound was at least one cm deep and produced a very bad malodor. The patient was hospitalized for four weeks and lost 50 kg in weight. Previous treatments with sharp debridement, maggot therapy, and systemic antibiotics were ineffective for 1.5 months, and MGH therapy was started (Figure 2a). The wound was infected with *Pseudomonas aeruginosa*. Within two days after MGH therapy was started, the malodor disappeared, while the infection was resolved within one week. Granulation tissue was evident after four weeks, and the wound was completely healed after 32 weeks of MGH therapy (Figure 2b). The total material costs for L-Mesitran treatment were €381 (11 × 50 g Ointment, 28 × 20 g Ointment; 5 × 15 g Soft).

### 2.3. Case 3

**Figure 3:**
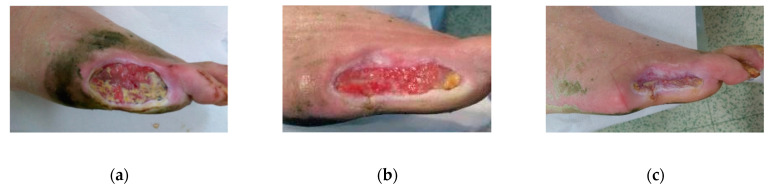
Case 3: (**a**) the wound at the start of MGH therapy (day 0); (**b**) progressed wound healing with healthy granulation tissue after three weeks of MGH therapy; (**c**) advanced healing of the wound after nine weeks of MGH therapy.

A 64-year-old male patient with type 2 DM presented to the wound clinic with a DFU on his right foot. The wound was previously treated with maggots without success for 15 days, and he subsequently received MGH therapy. The wound with dimensions of 7 × 3 cm after amputation of the fourth and fifth digit was painful, red, infected, and produced exudate and bad odor (Figure 3a, day 0). The wound was colonized with a polymicrobial infection (*Escherichia coli*, *Enterococcus faecalis*, *Finegoldia magna*, and *Bacterroides thetaiotaomicron*) resistant to ampicillin and penicillin. The malodor disappeared within one week after MGH therapy was started, while the infection was resolved within three weeks. After three weeks, granulation tissue was evident, and the wound size decreased to 6.5 × 2.5 cm (Figure 3b). After six weeks, wound healing further progressed, and the size was 5 × 1.5 cm, while it was completely healed after ten weeks of MGH therapy (Figure 3c, week 9). The total material costs for L-Mesitran treatment were €71 (1 × 50 g Ointment and 7 pcs Hydro; 1 × 15 g Soft and 4 pcs Tulle).

### 2.4. Case 4

**Figure 4:**
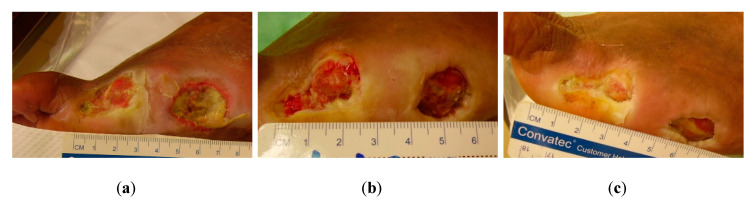
Case 4: (**a**) the wound at the start of MGH therapy (day 0); (**b**) resolution of infection and clear progression of wound healing after five weeks of treatment; (**c**) advanced healing of the wound after 11 weeks of MGH therapy.

A 52-year-old male patient with type 2 DM presented to the wound clinic with multiple wounds on his left foot. One DFU of 3 × 3 cm and one wound arose following amputation of his little toe three months ago. The patient was non-compliant with his diabetic medication and wound management, despite the foot being at risk of further amputation. The wound was ineffectively treated for three months with different wound care products, including hydrogel (Intrasite), silver dressings (Acticoat, Aquacel Ag), NaCl-gel (Hypergel), alginate dressings (Kaltostat), iodosorb powder, metronidazole (Flagyl), and papase. The wounds were infected with multi-resistant *Pseudomonas aeruginosa* bacteria (piperacillin/tazobactam and amoxicillin resistance). The malodor disappeared within a couple of days after MGH therapy was started, while the infection was resolved within three weeks. Granulation tissue was evident after three weeks, and, after five weeks, the wound size of the DFU decreased to 1.5 × 1.5 cm, a decrease of 50% (Figure 4b). Wound healing further progressed and was evident after 11 weeks of MGH therapy despite the patient being non-compliant to his therapies (Figure 4c). The total material costs for L-Mesitran treatment were €13 (1 × 50 g Ointment).

### 2.5. Case 5

**Figure 5:**
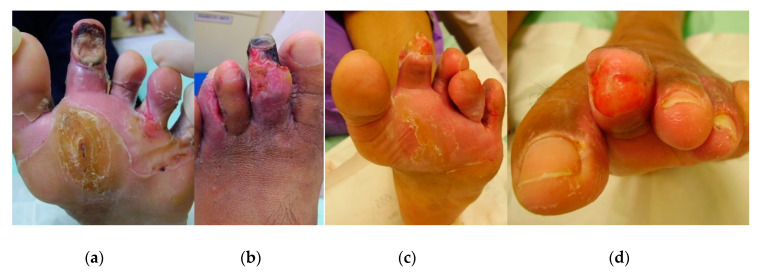
Case 5: (**a**) the wound at the start of MGH therapy (day 0); (**b**) gangrenous toe at day nine that needed amputation; (**c**) advanced healing of the wound after three weeks of MGH therapy; (**d**) wound closure after six weeks of MGH therapy.

A 45-year-old female patient with type 2 DM presented to the wound clinic with multiple wounds on the toes of her left foot. Due to the severity of the wound, there was a risk of amputation. Previous treatments included hydrogel (Intrasite), alginate (Kaltostat), film dressing (Melolin), and paraffin gauzes (Jelonet), all without success. The wounds were infected with multi-resistant (ampicillin and tetracycline) streptococci and *E. coli* bacteria (Figure 5a) and discharged pus. The malodor disappeared within a couple of days after MGH therapy started. After nine days, the damage of the toe was already so severe that an amputation of the gangrenous top part was unfortunately inevitable (Figure 5b). After three weeks of MGH therapy, the infection resolved, and granulation tissue was evident (Figure 5c), while the wound completely healed after six weeks (Figure 5d). The total material costs for L-Mesitran treatment were €13 (1 × 50 g Ointment).

### 2.6. Case 6

**Figure 6:**
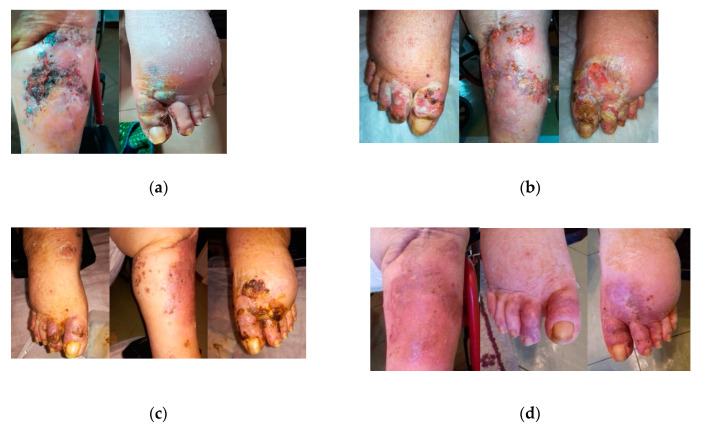
Case 6: (**a**) the wound at the start of MGH therapy (day 0); (**b**) MGH therapy led to autolytic debridement of necrotic tissue at day 12; (**c**) after one month, the edema was reduced and wound healing progressed; (**d**) complete healing of the wounds after seven weeks of MGH therapy.

An 80-year-old obese female patient with type 2 DM presented to the wound clinic with multiple feet and leg ulcers accompanied by swelling of the lower extremities. The wounds were previously treated for 15 days with povidone iodine (Betadin), neomycin sulfate (Pulvo 47), and no coverage, without success. The wounds were infected with a methicillin-resistant *Staphylococcus aureus* and subsequently treated with MGH therapy (Figure 6a, day 0). The malodor disappeared within a couple of days after the start of MGH therapy, while the infection resolved, and necrotic tissue was fully autolytic debrided within two weeks (Figure 6b, day 12). In week three, the wound showed healthy granulation tissue. After one month, edema and the wound size strongly reduced (Figure 6c). After seven weeks of MGH therapy, the diabetic ulcers were completely healed (Figure 6d). MGH treatment prevented a possible amputation and restored the quality of life for the patient. The total material costs for L-Mesitran treatment were €121 (1 × 50 g Ointment and 14 pcs Net; 1 × 50 g soft and 7 pcs Tulle).

The key observations of the presented cases are summarized in Table 1. On average, MGH controlled infection in 2.6 weeks. The formation of healthy granulation tissue occurred with an average of 3.5 weeks. Factors such as noncompliance, comorbid complications, and weight loss possibly compromised the healing trajectory in cases 3, 4, and 5. With time, all wounds, including these three, progressed toward healing, and amputation could be avoided in all cases.

## 3. Discussion

Diabetic patients with advanced wounds, such as presented in this case series, typically do not take good care of themselves and have bad hygiene. In addition, they have different comorbidities, such as obesity, hampered blood flow, atherosclerosis, neuropathy, and a bad nutritional diet, which attenuate the wound healing, making them even more prone to developing infections. Due to these comorbidities, these patients do not sense the pain at the beginning of a wound. When stayed unnoticed, wounds will worsen and get infected, especially with a lack of hygiene, such as walking in dirty bathrooms on bare feet and inadequate or no cleaning and wound care. Regular pedicure visits may prevent the development of wounds. However, when wounds are present, MGH may be a potent treatment strategy that should be considered more often as first-line therapy.

As presented in the current case series, whereas the previous therapies proved ineffective in treating DFUs, the application of MGH effectively enhanced wound repair. Within days, the malodor of the wounds neutralized, and inflammation and infection were controlled after a couple of weeks, including those with antibiotic-resistant bacteria. MGH holds multiple antimicrobial mechanisms that are very effective in resolving infections and, therefore, must be considered as a complementary treatment for antibiotics, especially since no resistance will be developed toward MGH. In addition to the antimicrobial activity, MGH also enhances wound healing. Here, MGH clearly enhanced autolytic debridement, leading to quick elimination of slough and the appearance of healthy granulation tissue within the following weeks. Due to the underlying pathologies, healing time ranged from 1.5–8 months.

Chronic wounds may be arrested in an inflammatory phase due to an ongoing infection or chronic inflammatory or oxidative stress in the wound bed. MGH can create a switch in the micro-environment of the wound by eradicating the bacterial load and changing the wound physiology due to its anti-inflammatory and anti-oxidative activities. Moreover, MGH accommodates a moist wound environment, has a low pH, and modulates oxygen levels, osmotic pressure, and proteinase activities that can cause non-healing wounds to suddenly start healing.

Edema, such as experienced in cases 1 and 6, can increase pressure on the wound, and it often leads to pain and a decreased quality of life for the patients. Infected wounds typically produce a lot of exudate, being presented as wet wounds. MGH consists of about 80% sugars (glucose, fructose, and sucrose), and these have a hygroscopic activity, attracting fluid from their environment. In wounds, this helps to draw out lymph fluid and clean the wound, stimulates autolytic debridement to remove necrotic tissue and slough, and decreases edema, as demonstrated in cases 1 and 2. The osmotic activity of MGH can result in elevated production of exudate, which may sometimes be noticed during wound dressing changes, especially at the beginning of MGH therapy when the MGH switches the wound environment.

Venous insufficiency will limit the transport of leucocytes and nutrition to the wound site, which is necessary to fight infections and promote healing. MGH will replace the task of leucocytes via its antimicrobial activity, and it can serve as an important nutrient source that is needed for proliferation and migration of epithelial cells during the re-epithelialization process [19,20].

The efficacy and the safety of MGH for the treatment of DFUs were often investigated. Observational studies demonstrated that MGH can be used safely [21]. Makhdoom et al. observed excellent results with natural honey in 14 diabetic wounds, and the disability of these patients was minimized by decreasing the rate of leg or foot amputations [22]. A larger study in 172 diabetic patients also showed that the use of honey significantly reduced the amputation rate and improved wound healing in chronic diabetic foot ulcers [23]. Moghazy et al. found that commercial clover honey is a clinical and cost-effective dressing for diabetic wounds in developing countries [24]. In the systematic review and meta-analysis of Wang et al., MGH treatment shortened the wound debridement time, wound healing time, and bacterial clearance time and increased the wound healing rate and bacterial clearance rate during the first 1–2 weeks of use [25]. Many different MGH formulations exist, andm since honey is a natural product, there is a difference in antimicrobial and healing activities.

A direct in vitro comparison study of L-Mesitran Soft and Medihoney against multiple (antibiotic-resistant) strains of staphylococci and *Pseudomonas* spp. pathogens showed that L-Mesitran had a more robust antimicrobial activity, despite containing half the concentration of honey [11]. This may have been caused by the difference in the type of honey, as there can be a 100-fold difference in antimicrobial activity between honey types [26]; alternatively, it may have been caused by the supplements added to L-Mesitran Soft [11]. Other studies demonstrated that L-Mesitran Soft has a stronger antimicrobial activity than its raw honey, which supports that the supplements added to the formulation, such as vitamins C and E, enhance the antimicrobial activity [11,12,13].

The quality of life of patients with DFUs is negatively affected. The malodor is unpleasant and may limit people who want to visit; additionally, the wound may heavily exudate that may stain clothes and require extra care, or these wounds with or without edema may be excruciating. MGH will attenuate all these problems and improve the quality of life for patients suffering from (infected) DFUs. MGH offers an alternative nutrient source for the bacteria, which switch from catabolizing smelling tissue debris and proteins to the odorless consumption of glucose, which subsequently rapidly decreases malodor. The high sugar content of MGH attracts lymph fluid and wound exudate out of the tissue into the wound dressing. This process, together with the anti-inflammatory activity of MGH, will subsequently reduce edema and pain. Diabetic patients with advanced wounds typically do not take good care of themselves and have bad hygiene. In addition, they have different comorbidities, such as obesity, hampered blood flow, atherosclerosis, neuropathy, and a bad nutritional diet, which attenuate the wound healing, making them even more prone to developing infections. These patients do not sense the pain of a beginning wound because of their neuropathy and do not adequately clean their wounds. Regular pedicure visits may prevent the development of wounds. However, when wounds are present, MGH may be a potent treatment strategy. In line with previous conclusions, we agree that MGH with its antimicrobial activity, via decreasing wound healing time and amputations and, thus costs, is a cost-effective treatment for the treatment of DFUs [22,23,24,25]. To illustrate, hospitalization costs for DFU patients needing an amputation range from United States dollars (USD) $12,851 to USD $16,267 [27]. By reducing the number of chronic wounds, not only will the wound care and societal costs decrease, but the quality of life for the patients will also be substantially improved [27,28].

In this case series, the products were easy to apply and provided excellent patient comfort. MGH prevents the adherence of newly formed granulation tissue into the wound dressing and does not re-open the tissue after removal; therefore, dressings can be replaced without pain. The high sugar content of the honey did not influence the blood glucose levels of the patients.

## 4. Materials and Methods

Diabetic ulcers were treated with one or more MGH formulations of the product range of L-Mesitran (Soft, Ointment, Net, Tulle, or Hydro, manufactured by Triticum Exploitatie BV, Maastricht, the Netherlands).

### 4.1. Subjects and therapeutic interventions

Following a multicenter approach, we included six patients with infected diabetic ulcers, of which five were colonized with antibiotic-resistant bacteria. Three patients had previous amputations, and, in four cases, there was a risk of (further) amputation. A plethora of prior treatments, including antibiotics, silver and alginate dressings, surgical closure, and maggot therapy, were ineffective. Subsequently, the treatment was switched to the application of MGH as monotherapy in all cases except one in which systemic antibiotics had to be administered as part of the hospital regulations. Wounds were cleaned following the local cleaning protocol. Compression therapy was temporarily given in cases 1 and 6 until the edema decreased, and only one patient (case 1) received medication (lisinopril 20 mg/day) to lower blood pressure. Patients were advised to offload the DFU to minimize pain, e.g., by using crutches during walking. The decision to use the L-Mesitran wound care product was dependent on the wound, the patient, and the experience of the wound care specialist. The products were applied following the manufacturer’s instructions and covered with a suitable secondary dressing. The dressing changes were done at the clinic, or the patients were clearly instructed to perform the dressing changes at home. An overview of the exact treatment protocol can be found in Table 2. Photos of the wounds were frequently taken to monitor and demonstrate the progression. All patients received medication to control their diabetes, and glucose levels were constantly monitored. No effect of L-Mesitran on blood glucose levels was observed.

The patients were informed about the study, and they all gave written informed consent to participate in the study and publication of the data. The principles of the World Medical Association’s Declaration of Helsinki were followed.

### 4.2. About L-Mesitran wound care products

L-Mesitran was the first MGH-based product to obtain both Food and Drug Administration (FDA) and European Conformity (CE: Conformité Européenne) approval in 2002 [29]. At that time, the initial scientific literature reported good antimicrobial activity for honey and showed that it could be effective for wound healing [7,30,31]. L-Mesitran manufactures a wide range of MGH-based wound care products, which can be used for different types of wounds. L-Mesitran Ointment and L-Mesitran Soft are a cream and gel, respectively. In general, the Ointment is a little thicker and contains slightly more MGH (48% versus 40% in L-Mesitran Soft), making the Soft a little easier to apply in deeper wounds. The more advanced wound care products of L-Mesitran are L-Mesitran Net, L-Mesitran Tulle, L-Mesitran Hydro/Border/Active, and the recently launched L-Mesitran Foam. In some of the presented cases, multiple L-Mesitran products were combined. L-Mesitran Net is a hydrogel that contains 20% MGH and can be used on moderate to heavily exuding wounds. However, it can also serve as a contact layer that stays well in place and, when necessary, can be combined with the L-Mesitran Ointment or L-Mesitran Soft for deeper wounds, which need a secondary dressing. L-Mesitran Tulle is a synthetic sterile gauze impregnated with L-Mesitran Soft and can be used on different kinds of wounds, for example, on superficial wounds; however, since it is so conformable, it can be easily applied to deeper wounds as well. The L-Mesitran Hydro, Border, and Active are all hydrogels that differ in size and having a border. They all contain 30% MGH and can absorb and encapsulate about 10 times their weight in wound fluid and, hence, can be used on exuding wounds. In addition, the instant cooling effect also makes them ideal for treating burn wounds. The newest product is L-Mesitran Foam, a highly absorbable foam dressing impregnated with L-Mesitran Soft, indicated for heavily exuding wounds. The polyurethane foam forms a cushioning layer that protects the wound from mechanical stress and allows drainage to pass, while the L-Mesitran Soft layer fights infections and optimizes wound healing.

## 5. Conclusions

In light of the rise in diabetes prevalence and complications related to DFUs, as well as increased antibiotic resistance, it is essential to explore novel wound treatment options. MGH forms a potent strategy to fight (antibiotic-resistant) infections and serves as a promising alternative antimicrobial chemotherapeutic without a risk of developing resistance. MGH is easy to apply in clinic and home care, and it is proven to be a safe and cost-effective treatment for chronic diabetic ulcers. The application of MGH resolves infections and enhances wound healing, and it forms a promising strategy as the first line of therapy in diabetic ulcers, as well as other type of wounds.

## Figures and Tables

**Table 1:** Wound healing trajectory per case, including time to full healing, time to reduce inflammation, and infection control.

Case	1	2	3	4	5	6
t _Infection_	4 weeks	1 week	3 weeks	3 weeks	3 weeks	2 weeks
t _Granulation_	4 weeks	4 weeks	3 weeks	3 weeks	3 weeks	3 weeks
t _Healing_	52 weeks	32 weeks	10 weeks	12 weeks	6 weeks	7 weeks
Risk _Amputation_		Yes	Yes	Yes	Yes	

**Table 2:** Wound management overview of each case and their respective L-Mesitran products. Ointment, a 48% MGH lanolin ointment; Soft, a 40% MGH wound gel; Net, a hydrocolloid net dressing containing 20% MGH; Hydro, a hydrogel containing 30% MGH.

Case	Initial 1° Dressing	Initial 2° Dressing	Duration and Dressing Regime	1° Dressing Treatment Change	2° Dressing Treatment Change	Duration and Dressing Regime
1	Soft and Net	Sterile absorptive gauzes	4 weeks of daily changes	Soft	Sterile absorptive gauzes	48 weeks of daily changes following weekly application
2	Ointment and Net	Sterile absorptive gauzes	2 months of two changes per day	Soft	Sterile absorptive gauzes	6 months of daily changes
3	Ointment and Hydro	Fixative gauzes	7 weeks of daily changes	Soft and Tulle	Sterile absorptive gauzes	3 weeks of daily changes
4	Ointment	Film dressing	2 weeks of daily changes	Ointment	Alginate and film dressing	10 weeks of daily changes
5	Ointment	Film dressing	6 weeks of daily changes			
6	Ointment and Net	Sterile absorptive gauzes	3 weeks of daily changes	Soft and Tulle	Sterile absorptive gauzes	3 weeks of daily changes

## References

[1] Cho N.H., Shaw J.E., Karuranga S., Huang Y., da Rocha Fernandes J.D., Ohlrogge A.W., Malanda B. (2018). IDF Diabetes Atlas: Global estimates of diabetes prevalence for 2017 and projections for 2045. Diabetes Res. Clin. Pract..

[2] Wild S., Roglic G., Green A., Sicree R., King H. (2004). Global prevalence of diabetes: Estimates for the year 2000 and projections for 2030. Diabetes Care.

[3] Baena-Diez J.M., Penafiel J., Subirana I., Ramos R., Elosua R., Marin-Ibanez A., Guembe M.J., Rigo F., Tormo-Diaz M.J., Moreno-Iribas C. (2016). Risk of Cause-Specific Death in Individuals With Diabetes: A Competing Risks Analysis. Diabetes Care.

[4] van Netten J.J., Bus S.A., Apelqvist J., Lipsky B.A., Hinchliffe R.J., Game F., Rayman G., Lazzarini P.A., Forsythe R.O., Peters E.J.G. (2020). Definitions and criteria for diabetic foot disease. Diabetes Metab Res. Rev..

[5] Brem H., Tomic-Canic M. (2007). Cellular and molecular basis of wound healing in diabetes. J. Clin. Investig..

[6] Smaropoulos E., Cremers N.A.J. (2020). Treating severe wounds in pediatrics with medical grade honey: A case series. Clin. Case Rep..

[7] Molan P.C. (2002). Re-introducing honey in the management of wounds and ulcers – theory and practice. Ostomy Wound Manage..

[8] Hermanns R., Mateescu C., Thrasyvoulou A., Tananaki C., Wagener F.A., Cremers N.A. (2020). Defining the standards for medical grade honey. J. Apic. Res..

[9] Postmes T., van den Bogaard A.E., Hazen M. (1993). Honey for wounds, ulcers, and skin graft preservation. Lancet.

[10] Maddocks S.E., Jenkins R.E. (2013). Honey: A sweet solution to the growing problem of antimicrobial resistance?. Future Microbiol..

[11] Cremers N., Belas A., Santos Costa S., Couto I., de Rooster H., Pomba C. (2020). In vitro antimicrobial efficacy of two medical grade honey formulations against common high-risk meticillin-resistant staphylococci and Pseudomonas spp. pathogens. Vet. Dermatol..

[12] Hermanns R., Cremers N.A.J., Leeming J.P., van der Werf E.T. (2019). Sweet Relief: Determining the Antimicrobial Activity of Medical Grade Honey Against Vaginal Isolates of Candida albicans. J. Fungi (Basel).

[13] Oliveira A.M.P., Devesa J.S.P., Hill P.B. (2018). In vitro efficacy of a honey-based gel against canine clinical isolates of Staphylococcus pseudintermedius and Malassezia pachydermatis. Vet. Dermatol..

[14] Saikaly S.K., Khachemoune A. (2017). Honey and Wound Healing: An Update. Am. J. Clin. Dermatol..

[15] Gottrup F. (2004). Oxygen in wound healing and infection. World J. Surg..

[16] Rossiter K., Cooper A.J., Voegeli D., Lwaleed B.A. (2010). Honey promotes angiogeneic activity in the rat aortic ring assay. J. Wound Care.

[17] Winter G.D. (1962). Formation of the scab and the rate of epithelization of superficial wounds in the skin of the young domestic pig. Nature.

[18] Lukanc B., Potokar T., Erjavec V. (2020). Complete skin regeneration with medical honey after skin loss on the entire circumference of a leg in a cat. J. Tissue Viability.

[19] Du Toit D.F., Page B.J. (2009). An in vitro evaluation of the cell toxicity of honey and silver dressings. J. Wound Care.

[20] Smaropoulos E., Cremers N.A. (2020). Medical grade honey for the treatment of paediatric abdominal wounds: A case series. J. Wound Care.

[21] Kateel R., Adhikari P., Augustine A.J., Ullal S. (2016). Topical honey for the treatment of diabetic foot ulcer: A systematic review. Complement. Ther. Clin. Pract..

[22] Makhdoom A., Khan M.S., Lagahari M.A., Rahopoto M.Q., Tahir S.M., Siddiqui K.A. (2009). Management of diabetic foot by natural honey. J. Ayub Med. Coll Abbottabad.

[23] Surahio A.R., Khan A.A., Farooq M., Fatima I. (2014). Role of honey in wound dressing in diabetic foot ulcer. J. Ayub Med. Coll Abbottabad.

[24] Moghazy A.M., Shams M.E., Adly O.A., Abbas A.H., El-Badawy M.A., Elsakka D.M., Hassan S.A., Abdelmohsen W.S., Ali O.S., Mohamed B.A. (2010). The clinical and cost effectiveness of bee honey dressing in the treatment of diabetic foot ulcers. Diabetes Res. Clin. Pract..

[25] Wang C., Guo M., Zhang N., Wang G. (2019). Effectiveness of honey dressing in the treatment of diabetic foot ulcers: A systematic review and meta-analysis. Complement. Ther. Clin. Pract..

[26] Mandal M.D., Mandal S. (2011). Honey: Its medicinal property and antibacterial activity. Asian Pac. J. Trop. Biomed..

[27] Olsson M., Jarbrink K., Divakar U., Bajpai R., Upton Z., Schmidtchen A., Car J. (2019). The humanistic and economic burden of chronic wounds: A systematic review. Wound Repair Regen..

[28] Jarbrink K., Ni G., Sonnergren H., Schmidtchen A., Pang C., Bajpai R., Car J. (2017). The humanistic and economic burden of chronic wounds: A protocol for a systematic review. Syst. Rev..

[29] Zbuchea A. (2017). Honey, Food and Medicine: Scientific Rationale and Practical Efficiency in External Administration of Medicinal Honey for Wound Healing. J. Agric. Sci. Technol. B.

[30] Moore O.A., Smith L.A., Campbell F., Seers K., McQuay H.J., Moore R.A. (2001). Systematic review of the use of honey as a wound dressing. BMC Complement. Altern. Med..

[31] Lusby P.E., Coombes A., Wilkinson J.M. (2002). Honey: A potent agent for wound healing?. J. Wound Ostomy Continence Nurs..

